# Traits plasticity of Sodom Apple (*Calotropis procera*) along the environmental gradient in the semi-arid environment

**DOI:** 10.3389/fpls.2022.1047632

**Published:** 2023-02-08

**Authors:** Siraj, Nasrullah Khan, Mohammad Okla, Saud Al-amri, Wahidah Al-Qahtani, Mostafa Abdel-Maksoud, Hamada AbdElgawad

**Affiliations:** ^1^ Department of Botany, University of Malakand, Chakdara, Khyber Pakhtunkhwa, Pakistan; ^2^ Department of Botany and Microbiology, College of Sciences, King Saud University, Riyadh, Saudi Arabia; ^3^ Department of Food Sciences & Nutrition, College of Food & Agriculture Sciences, King Saud University, Riyadh, Saudi Arabia; ^4^ Integrated Molecular Plant Physiology Research (IMPRES), Department of Biology, University of Antwerp, Antwerp, Belgium; ^5^ Zoology Department, College of Sciences, King Saud University, Riyadh, Saudi Arabia

**Keywords:** biomass variations, environmental factors, allometric equation, morphological traits, Sodom apple

## Abstract

Biomass and morphological characteristics of plant species provide essential insight into how well a species adapts to its environment. The study aims to evaluate how environmental variables (viz., altitude, slope, aspect degree, and soil properties) influence the morphological traits and biomass variability of *Calotropis procera* (Aiton) W.T. Aiton in a semi-arid environment. *C. procera* sample locations were divided into 39 permanent sites (5×5 = 25 m^2^). Slope, aspect degree, slope aspect, altitude, and soil variables (soil moisture, organic matter, nitrogen (N %), and phosphorus (P) gradients were used to quantify morphological parameters (height, diameters, canopy area, volume, and leave/branch biomass) and aboveground biomass. Environmental variables, i.e., altitude and aspect degree, were the most important factor influencing the biomass variation and affecting soil moisture content; however, they did not directly affect the total biomass of the species. The results also reveal significant plasticity in morphological traits exists concerning elevation and aspect degree at (*p<* 0.05). Plant volume was a better indicator of species’ total biomass revealed from the regression model showing significant at *p<* 0.05. The study also reveals that soil properties such as soil moisture and Phosphorus have an important role in enhancing the productivity of the studied plant species. The results concluded that plants functional traits and biomass shows significant variation across the altitude and these parameters could be consider in the conservation of this native species.

## Introduction

The link between environmental factors and vegetation has long attracted ecologists (Dearborn and Danby, 2017; Sanaei et al., 2018). At the regional scale, topography and climatic differences produce substantial spatial variety in plants and soil (Lybrand and Rasmussen, 2015). Slope, slope aspect, and slope location, among other environmental elements, may considerably influence plants on a local scale by altering radiation, temperature, water, and nutrients (Dearborn and Danby, 2017). It is widely accepted that biotic (species interactions and species-specific characteristics), abiotic (topography, climate, soil, etc.), and anthropogenic (over-exploitation, deforestation) factors all affect biodiversity and ecosystem processes, resulting in complex interactions (Hooper et al., 2005; Schumacher and Roscher, 2009). Topographic characteristics, such as height, slope, solar aspect, interactions with other species, and dispersion capacities, impact the distribution and abundance of a plant species (Rahman et al., 2022).

Plant ecological strategies, species abundance, coexistence mechanisms, community assembly, and the ecological consequences and responses of plant communities to their environment may be described and predicted using morphological distinctions (Ullah et al., 2021). Ecosystem processes may be influenced in two ways: directly by modifying ecosystem flux rates of energy and matter or indirectly by affecting plants’ physiological rates, which are based on functional features of the plants (Hajek et al., 2016). Plant biomass may be connected to biological and abiotic filters (Díaz et al., 2007). As an example, plant fitness and survival, and biomass production may be affected by functional characteristics (Loiola et al., 2015). Furthermore, functional characteristics may show how a species interacts with and responds to its environment, making them a beneficial tool for solving ecological concerns (McGill et al., 2006). Ecosystem processes may be influenced in two ways: directly by modifying ecosystem flux rates of energy and matter or indirectly by affecting plants’ physiological rates, which are based on functional features of the plants (Hajek et al., 2016). Plant biomass may be connected to biological and abiotic filters (Díaz et al., 2007). As an example, plant fitness and survival, and biomass production may be affected by functional characteristics (Loiola et al., 2015).

Therefore, allometric equations are often used in the estimation of shrub biomass (Arias et al., 2011). Allometric models rely on connections between biomass and morphological features such as stem diameter and plant height (Kuyah et al., 2016). The biomass of certain species varies depending on terrain, ambient conditions, stand ages, species composition, and natural and human-induced disturbances, generalized formulae are ineffective (Melson et al., 2011). In addition, species-specific and site-specific allometric models are the best for lowering biomass estimate uncertainty (Hossain et al., 2016) and suitable indicators of plant functional traits variation across the environmental gradient.

In the Asclepiadaceae family, *Calotropis procera* (Aiton) is an evergreen perennial shrub that appears in broad climate ranges and reproduces mainly by seed (Hassan et al., 2015). The plant grows only in arid and semiarid regions of Asia and Africa (Lottermoser, 2011). Diversity and adaptation capacity of species have a significant impact on population dynamics (Rahman et al., 2021). Unfortunately, a lack of accurate biomass estimates for this valuable species caused problems in ecosystem and habitat assessments since shrubland stand management was based on growing plants. In addition, the species is under an extremely high extinction threat in Pakistan as a direct result of unchecked and excessive collecting efforts by locals for medical and commercial use.

Therefore, the objective of this study is to establish an allometric equation specific to *C. procera* biomass based on values of plant height, canopy cover, plant volume, and plant components, like root stems and leaf fruit, with the assumption that these factors can be used to model biomass specific to *C. procera*. Moreover, the functional properties of *C. procera* in abiotic environments were investigated to see whether the species’ high population density, biomass, and morphological and reproductive traits contribute to its capacity to thrive in a variety of microhabitats. This research will also aid in increasing the species’ biomass in light of current environmental conditions and improved propagation for conservation reasons.

## Materials and methods

### Study area

**Figure 1:**
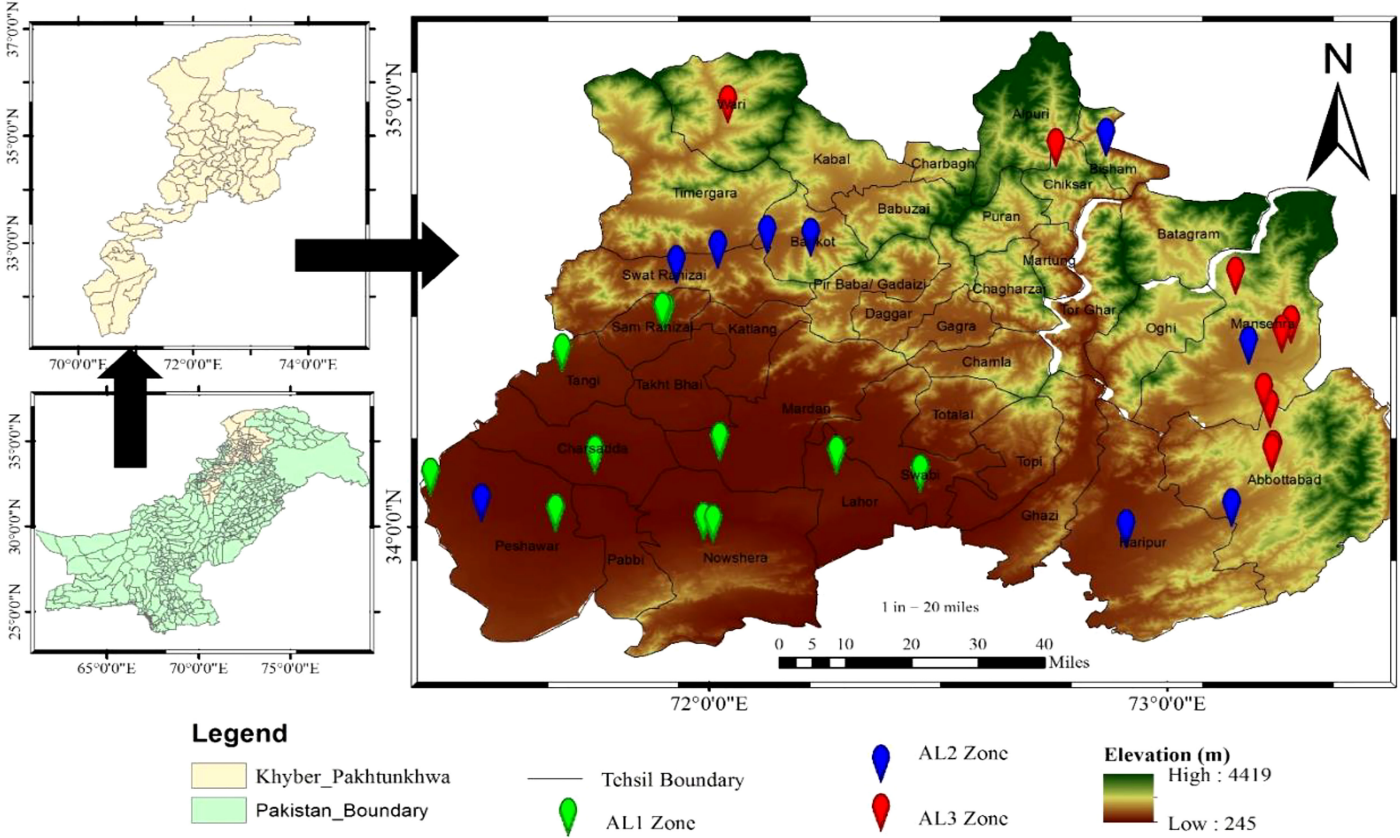
Study area map showing distribution of the studied plant along different elevation.

The sample area was in Khyber Pakhtunkhwa, Pakistan, with latitude of 34.9526 °N and a longitude of 72.3311 °E (**Figure 1**), with a total geographical area of approximately 101,741 km^2^.For the phytosociological data collection, thirty-nine (39) sites were selected across three elevational gradients (high elevation = (AL 3) > 1420 m a.s.l; medium elevation = (AL 2) > 1256 m a.s.l; and low elevation = (AL 1) > 331 m a.s.l). The field survey was conducted from February 2018 to May 2020. The research locations’ latitude, longitude, and aspect degree were recorded using a GPS, and the slope was assessed using a clinometer. The average annual precipitation was 400 mm, with the lowest and highest temperatures of 2°C and 40°C, respectively. Within the thirty-nine permanent quadrats (5×5 = 25 m^2^) used to measure biomass, they found that members of the species were healthy, disease-free, and devoid of physical injury. A composite soil sample was collected from each site from the plant’s root zone, i.e., 50-80cm depth. The samples were sorted into three categories for floristic data based on altitudinal zonation and delivered to the Swat Agriculture Research Institute (ARI). Soil extract was used to determine Organic matter, Potassium (K), Nitrogen (N), and Phosphorus (P) (Rhoades, 1993; Nelson and Sommers, 1996). Available water was calculated by using an online calculator developed by Saxton and Rawls (2006).

### Field data collection

Each quadrat was tagged with different colours to calculate the density of *C. procera* (i.e., the number of individuals per unit area). The leaf area was measured using the trace paper method, and the leaves/branches were randomly counted by picking five separate branches. Similarly, the standard procedure was used to compute plant diameter, height (m), and volume (Pandey and Singh, 2011). Plant height and diameter ratio fluctuate in different microhabitats. Hence these characteristics were considered when estimating shrub size (Shaltut and Ayyad, 1990). To calculate dry biomass, 39 complete plants were dug up, and each portion (stem, leaf, and root) was preserved individually in polythene bags. The destructive approach was used to calculate the plant biomass (Wang et al., 2008). All of the samples were oven-dried for 48 hours at 65 degrees Celsius. The dry weight was obtained by weighing the samples after being returned to the Department of Botany at the University of Malakand in KP, Pakistan. Based on the data of the plant crown diameter (C), we calculated the canopy area (CA m^2^) and volume (V m^3^) using the techniques given by Zeng et al. (2010).


(1) $$CA=\frac{C2\;}{\text{π}\times 3.14}$$

(2) V=CA×H

Where CA stand for canopy area, V for volume and H for height of studied plant.

### Allometric equation and biomass calculation

A regression equation was developed in order to determine the association between shrub total biomass, used as the dependent variable, and other growth characteristics such as diameter, height, height-to-diameter ratio, canopy area, and volume (D^2^, H, D^2^H, CA, and CV), used as the explanatory factors.


(3) Linear model:y=a+bx

To determine whether the regression equations were accurate representations of the data, the coefficient of determination (R^2^), standard error estimate (SEE), and F-values between the pairs of x and y variables were calculated (Zeng et al., 2010).

### Statistical analysis

Analysis of variance (ANOVA) and Tukey HSD *post hoc* tests was employed to identify significant differences in the biomass ratio and the other variables evaluated. To estimate growth parameters and biomass, an allometric regression equation was applied. In order to determine if the biomass and other growth metrics exhibited significant responses, a permutational multivariate analysis of variance (PERMANOVA) was performed, and ordinations were formulated. This approach is thought to be particularly well adapted to ecological data, especially when working with a small sample size (Andringa et al., 2019). The “Adonis” technique in the vegan package 1.13-2 was utilized in the PERMANOVA with the Euclidean distance approach (Sabo et al., 2019; Oksanen et al., 2020). Non-metric multidimensional scaling (NMDS) was used to depict the ordinals, utilizing the default parameters of vegan’s “meta MDS” method (Kruakal, 1964). A total of 999 permutations were employed in this investigation. The statistical analysis was carried out using R software using the dplyr, tidyverse, ggplot2, and vegan packages.

## Results

### Environmental variables and plant traits

**Table 1** summarises the spectrum of characteristics and biomass changes of *Calotropis procera* throughout the three altitudinal groups. There were no significant differences in total biomass between various slope locations and slope angles. Plant populations may be distinguished by physical traits, such as stem height (2.63 ± 0.21 m) and crown diameter (2.18 ± 0.27 m) for those found at high elevations (AL 3). *C. procera* grows well in disturbed and open environments. Hence individuals reported from rural regions with low altitude (AL 1) were shorter (H=1.66 ± 0.13 m, D=1.99 ± 0.13 m) (**Table 1**). The H/D ratio was larger than one in all habitat categories, with urban sites with high altitude (AL 3) have the greatest ratio (1.21 ± 1.95 m) and rural sites having the lowest (0.83 ± 0.73 m). There was a considerable fluctuation in the height of the stem, diameter, size index, and volume of *C. procera* in the current study, indicating the species’ growth adaptability in disturbed habitats. The biomass of the investigated plant was high at a high altitude (79.85 ± 4.90) in the sample locations but exhibited heterogeneity in all groups.

**Table 1:** Summary of morphological traits and stand description (Mean ± SE) of sample habitats and biomass component for *C. procera*.

Variables	AL 1	AL 2	AL 3	F-value
Altitude	564.3 ± 142^b^	1055.2 ± 126^c^	1377.6 ± 70.6^a^	6.28**
Slope degree	154.8 ± 26.8^a^	181.6 ± 26.5^b^	161.1 ± 39.1^b^	4.28*
Average plant height (m)	1.66 ± 0.13^a^	2.12 ± 0.11^b^	2.63 ± 0.21^c^	10.01***
Average diameter (mm)	1.99 ± 0.13^a^	1.62 ± 0.12^a^	2.18 ± 0.27^b^	6.64**
H/D	0.83 ± 0.73^a^	1.31 ± 1.02^a^	1.21 ± 1.95^b^	12.35***
CA (m²)	3.18 ± 0.28^b^	2.23 ± 0.32^a^	3.25 ± 0.35^b^	6.34**
CV (m³)	9.88 ± 0.75^b^	4.81 ± 0.79^a^	5.12 ± 0.57^a^	12.35***
Leave/branch	13.25 ± 0.79^a^	23.67 ± 2.19^b^	36.16 ± 2.28^c^	28.67***
Leaf area(cm^2^)	120.6 ± 48.7^a^	135.8 ± 73.9^b^	148.4 ± 68.6^c^	24.25***
Stem biomass (g)	41.72 ± 4.0^a^	43.83 ± 4.1^a^	45.66 ± 4.03^a^	2.25
leaf biomass (g)	29.48 ± 2.7^a^	31.13 ± 2.3^a^	30.10 ± 1.98^a^	3.25
Root biomass (g)	43.03 ± 4.4^a^	41.97 ± 4.2^a^	46.89 ± 3.87^a^	3.59
Aboveground biomass (g)	75.86 ± 7.2^a^	77.06 ± 6.5^a^	79.85 ± 4.90^a^	3.25
*OM %*	0.85 ± 0.13^a^	0.90 ± 0.29^a^	0.97 ± 0.22^b^	0.98
*N %*	0.09 ± 0.02^a^	0.04 ± 0.02^a^	0.19 ± 0.08^b^	0.343
*P (mg/Kg)*	4.46 ± 0.45^a^	4.37 ± 0.43^a^	4.45 ± 0.52^a^	0.716
*K (mg/Kg)*	90.33 ± 8.63^a^	85.625 ± 10.49^a^	111.57 ± 17.93^b^	1.388
*AW %*	0.13 ± 0.00^a^	0.13 ± 0.01^a^	0.13 ± 0.01^a^	0.941

CA, Canopy area; CV, Canopy volume; OM, organic matter; N, nitrogen percentage; K, Potassium; P, Phosphorous content; AW, Available water; AL1, Low altitude; AL2, Middle altitude; AL3, High altitude. * , P < 0.05; ** , P < 0.01; *** , P < 0.001.

**Figure 2:**
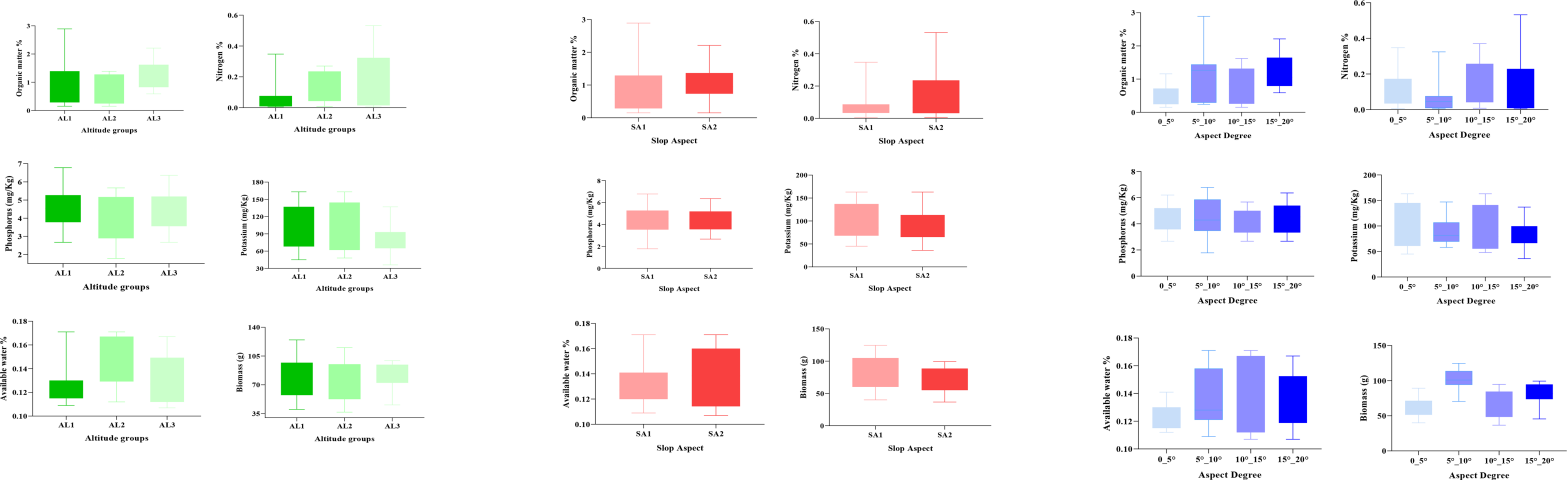
Relationship between topographic conditions and soil variables for *C. procera*. Error bars represent stand deviation. Low altitude (AL1), middle altitude (AL2), high altitude (AL3), Slope aspect (south-facing and North-facing slope), Aspect degree (0-5°, 5°-10°, 10°-15°, 15°-20°).

The standing effect of ground factors (slope, elevation, slope direction) on the biomass of *C. procera* shrubs and soil variables (N %, P mg/Kg, K mg/Kg, OM %, and AW %) using boxplots (**Figure 2**). The result declared that the soil variables (OM %, N %, P mg/Kg, K mg/Kg) and biomass of plant show a decreasing trend from low to high altitude except for available water contents (AW %) and Potassium (K mg/Kg) contents. **Figure 2** demonstrates that different stand factors significantly affect the distribution pattern, whereas when slope direction changes from north-facing slope (AS 1) to south-facing slope (AS 2), no other significant differences are noticed except for available water (AW %) and nitrogen content (N%). Furthermore, it had a significant increasing trend of slop from 0-5° to 15°-20° for all soil variables (**Figure 2**).

### Environmental variables influence trait plasticity

The influence of stand variables on various biomass and morphological features varied greatly, as shown in **Table 2**. The Euclidean distance for the distribution pattern of *C. procera* biomass and morphological traits was used to determine the PERMANOVA findings in this research. There were significant differences in all site parameters on biomass (*p<* 0.005). The biomass by site parameters had a considerable degree of explanation (R^2^) (*p*< 0.05). A greater impact of slope direction and elevation was observed on *C. procera* shrub biomass, such as for biomass, altitude (0.45)< aspect-degree (0.62)< slope-aspect (0.76); for height, altitude (0.73) > aspect-degree (0.39)< slope-aspect (0.73); for canopy area, altitude (0.63) > aspect-degree (0.25)< slope-aspect (0.82) and canopy volume, altitude (0.33)< slope-aspect (0.50)< aspect-degree (0.87). The volume was significantly affected by the interplay of height and aspect degree. The biomass distribution patterns of *C. procera* were significantly affected by the interaction of the three site variables. From **Table 2**, it can be seen that Euclidean distance can be used to calculate the PERMANOVA results for individual stand factors.

**Table 2:** Results of PERMANOVA of Biomass and Morphological traits with site conditions and their interactions for *C. procera*.

Site factors	Biomass			Height			Diameter			Canopy area (m^2^)			Canopy Volume (m^3^)		
	F	R^2^	P	F	R^2^	P	F	R^2^	P	F	R^2^	P	F	R^2^	P
Altitude	4.26	0.45	**0.02**	7.12	0.73	**0.001**	3.65	0.34	**0.03**	4.51	0.63	**0.005**	3.55	0.33	**0.02**
Slope-aspect	5.29	0.76	**0.01**	4.26	0.39	**0.027**	8.32	0.77	**0.002**	2.06	0.25	**0.06**	7.56	0.87	**0.03**
Aspect degree	3.11	0.62	**0.03**	8.82	0.70	**0.003**	5.60	0.50	**0.02**	9.23	0.82	**0.001**	5.60	0.50	**0.001**
Altitude × Slope-aspect	2.16	0.15	0.29	2.12	0.13	0.12	1.65	0.10	0.11	1.09	0.03	0.25	2.16	0.15	0.11
Altitude × Aspect degree	1.29	0.06	0.31	1.26	0.13	0.27	2.08	0.21	0.06	3.13	0.47	**0.02**	1.29	0.06	0.06
Slope-aspect × Aspect degree	1.71	0.02	0.09	1.18	0.03	0.33	4.34	0.70	**0.002**	9.23	0.82	0.001	8.32	0.77	**0.002**
Altitude × Slope-aspect × Aspect degree	1.21	0.12	**0.03**	5.16	0.72	**0.003**	5.60	0.55	**0.001**	1.23	0.02	**0.08**	5.60	0.55	**0.001**

F, ANOVA F-value; R^2^, Adjusted R^2^; P, ANOVA P-value. ** , P < 0.01; *** , P < 0.001.

### Relationship of topographic variables with soil properties and biomass

**Figure 3:**
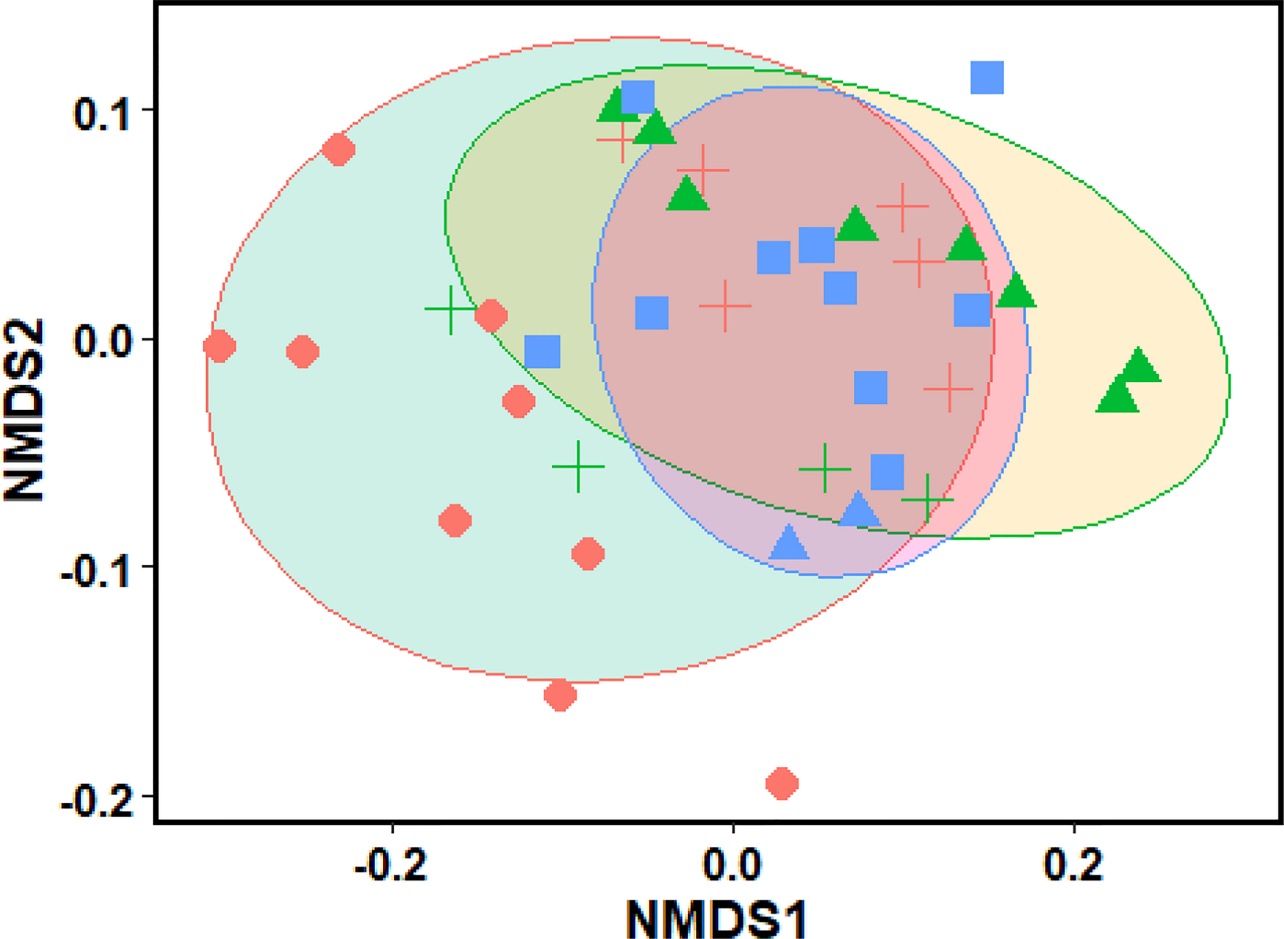
Distribution of sites in two-dimensional NMDS ordinations (stress = 0.04%) based on shrub environmental variables and trait plasticity for *Calotropis procera.* Ellipsis (Altitude), different symbols (biomass variation at different aspect degree), ellipsis color (slope aspect).

The biomass partitioning pattern of *C. procera* shrubs was mapped with environmental factors using the non-metric multidimensional scaling (NMDS) approach based on Euclidean distance. As illustrated in **Figure 3**, the distribution of loci within the two-dimensional NMDS classification scheme of *C. procera* shrub biomass can be seen. The NMDS sorting process may provide evidence of the link between shrub biomass and stand variables through limiting the number of axes and allowing the sorting axes to reflect particular environmental gradients. The findings of the PERMANOVA were confirmed by the NMDS ordination (stress = 0.04%), which demonstrated clustering of topographic effects on biomass allocation patterns, with the highest divergence between the variables (**Figure 3**). There were some changes in the distribution pattern of *C. procera* shrub biomass on various slope orientations and altitudes. The slope direction had the most significant changes.

### Biomass and allometric equations

**Table 3** shows the equation parameters, accuracy, and goodness equations established for the *C. procera* biomass estimate. *C. procera* was estimated using allometric equations across the three elevation groups. H, D, D^2^H, CA, and CV were used to see which growth characteristics predicted biomass most accurately. Based on linear modeling, the best fit equations for biomass estimate were derived using H, D, and CV as best predicted. These equations were statistically significant (*p*< 0.01). The linear equations showed the highest R^2^-value for flower biomass and total biomass except for the foliar biomass. Linear models with V, CA and H as independent variables showed the best fit for biomass of stem, leaves, and flower, respectively at rural areas (**Table 3**). However, D^2^ as independent variable had lowest R^2^ for total biomass. On the other side, at urban area stem biomass have high R^2^ value with respect to other organ biomass while at roadside flower biomass have high R^2^ value with V as independent variable.

**Table 3:** Linear model for *Calotropis procera* organ with biomass at different habitat.

Altitude	Organ	Variables	Lineary=a + bx	R²	SEE	F value
AL 1	Stem	X=V	y=7.7578x+18.3207	0.866	8.148	52.648**
	Leave	X=CA	y=18.4851x+3.6985	0.883	9.095	56.532**
	Flower	X=H	y=3.1383x+−2.5788	0.777	0.697	45.310
	Total ABG	X=D²	y=43.6740x+−3.5104	0.405	19.541	8.845
AL 2	Stem	X= D²	y=47.6185x−0.3788x	0.763	7.198	32.281
	Leave	X= CA	y=2.0879x+24.1057	0.196	6.471	2.637
	Flower	X=V	y=0.4653x+2.1889	0.271	1.684	3.718
	Total ABG	X= H	Y=19254x +49.646	0.154	18.41	1.82
AL 3	Stem	X= D²H	y=1.6375x+19.5264	0.697	8.145	23.020
	Leave	X= CA	y=1.6456x+11.9808	0.109	9.987	0.191
	Flower	X= V	y=0.4030x+0.3652	0.941	0.598	159.3***
	Total ABG	X=V	y=4.1346x+31.8724	0.873	9.323	69.033**

V (volume), CA (crown area CA= $\frac{\pi d^{2}}{4}$ ), D^2^ (crown diameter), H (height), Total ABG (Above ground biomass). AL1, Low elevation; AL2, Middle elevation; AL3, High Elevation; R^2^, Adjusted R^2^; **, P < 0.01; ***, P <0.001; SEE, standard error estimate.

**Figure 4:**
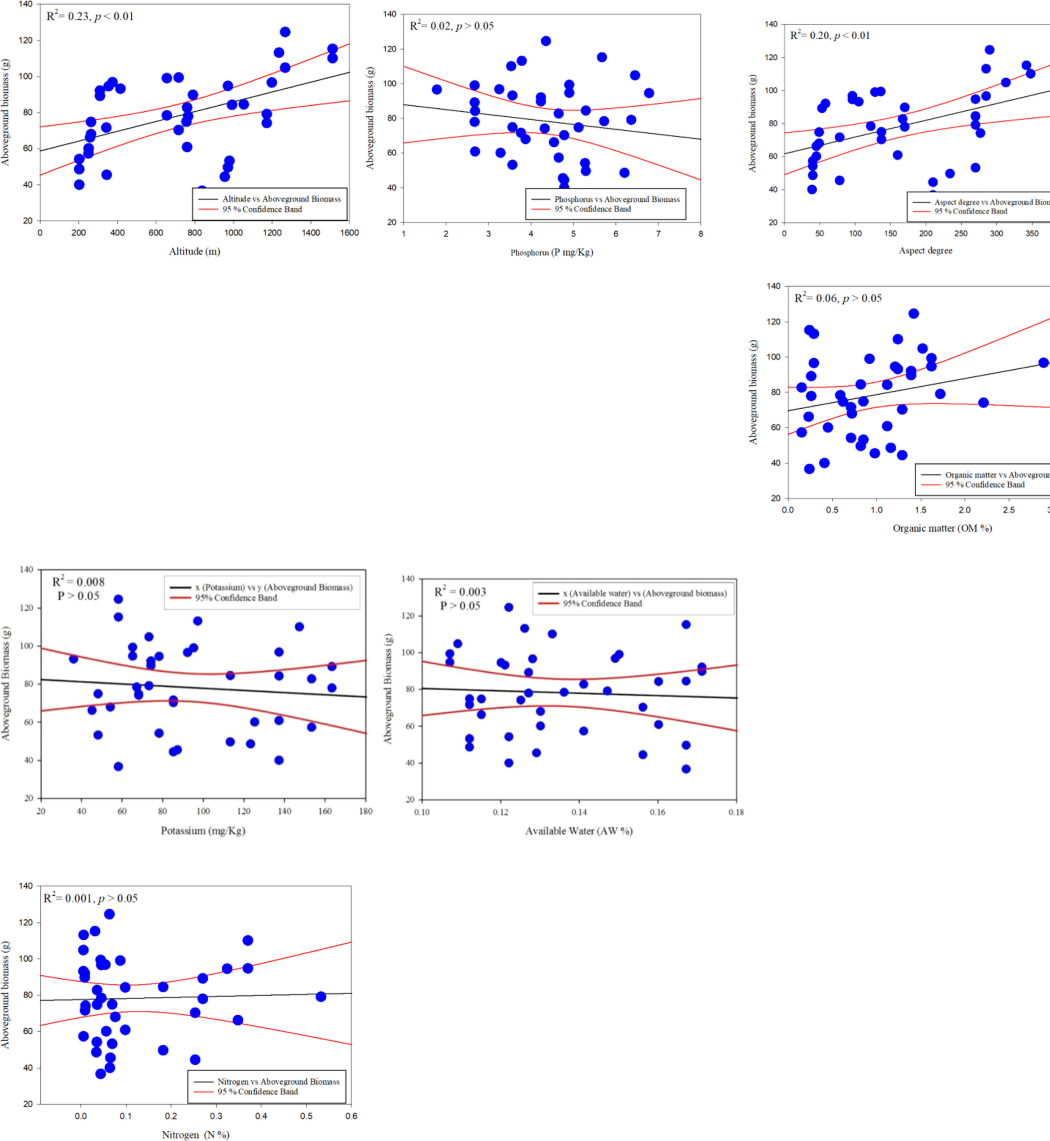
Linear regression model for *C. procera* biomass with environmental variables.

In contrast, stem biomass is linked to diameter and height at low elevations in urban and roadside environments (**Table 3**). The accuracy measurements suggest that one or two factors may adequately describe *C. procera* biomass (**Table 3**). There is, nevertheless, room to improve the model’s explanatory power by including more independent variables. D^2^, D^2^H, CA, and H are employed to analyse shrub biomass regressions as independent variables. The linear model was the best fit for providing a common statistical foundation for characterizing size–biomass connections in this investigation. H, D^2^H, and CV were the most relevant independent variables for this *C. procera* investigation. The linear regression model was used to show a significant relationship between the aboveground biomass in response to different environmental variables such as altitude, aspect degree, slope aspect, N %, P mg/Kg, K mg/Kg, OM %, and AW % (**Figure 4**).

## Discussions

*C. procera*, an important shrub, mainly reported from different parts of Pakistan, is under immense pressure due to natural and artificial activities (Kareem et al., 2008; Ahmad et al., 2021). Variation in the morphological characteristics was much visible along the different habitats occupied by this plant species. In the current study, we reported plants of *C. procera* to have stem height of 2.63 ± 0.3 m and crown cover of 2.18 ± 0.27 m which is comparatively higher than those reported from urban sites. This variation in size may be because the *C. procera* growth like open and disturbed areas (Sharma et al., 2012). Similarly, climatic and edaphic factors, interspecific competition, and standard density can also play a great role in determining the height and diameter of a plant species (Foroughbakhch et al., 2006). Variations in stem height, crown diameter, and volume are attributed to growth plasticity in diverse habitats (Hou et al., 2020).

Several workers have studied the importance of habitat characteristics on biomass allocation arrangement of plant species (Tang et al., 2021). Different environmental conditions coupled with edaphic factors are the key factors determining biomass (Li et al., 2013). The current study reported that slope angle highly influences the biomass allocation pattern, followed by elevation and aspect degree. Li and Xu (2019) documented the strong relation between slope degree and biomass of shrubby plants, which agrees with the findings of current results. Likewise, the report documented by Li et al. (2007) also highlighted a strong connection between altitude and slope angle toward biomass of different plant parts.

The elevation is a complex factor defining several other habitat conditions, including slope, light, precipitation, temperature, nutrients water availability (Ochoa-Gómez et al., 2019). Soil fertility and soil water availability are mainly controlled by slope angle and soil texture showing that plant growth will vary according to ecological factors (Wang et al., 2017), which supports the findings in the current study. Similarly, biotic factors like anthropogenic activities and interspecific and intraspecific competition can affect the biomass of plant species (Vieira et al., 2020). We reported significant-high biomass for the individuals positioned on the roadside more exposed to disturbances than those located in less disturbed areas. Therefore, it is concluded that the combined effect of environmental variables highly influences the biomass allocation pattern in plants (Yang et al., 2017).

In general, biomass allocation of shrubs is mainly attributed to leaf-mass, stem-mass, root-mass, and root to crown ratios. Different approaches have been adopted to measure the contribution of plant organs to biomass allocation. In the current study, we used PERMANOVA with Euclidean distance to determine plant parts shares to the total biomass following the standard protocols of Ochoa-Gómez et al. (2019). Euclidean distance has several advantages, including characterizing the actual distance among the points and applying it to the same property indicators. However, it cannot be applied to different indicators (Greenacre and Primicerio, 2014). A suitable method of calculating distance should be chosen based on the situation, as different methods have their advantages and disadvantages.

Different regression models have been developed to estimate shrub biomass, of which linear and quadratic models are more commonly used (Ali et al., 2022). A linear regression model was used to determine biomass in the current study, which provided a common statistical basis for analyzing size-biomass relationships.

We reported that the most appropriate independent variables were H, D/H, and CV, which supports the findings of Zeng et al. (2010). Due to the shrubby nature of *C. procera*, the current study used crown diameter for calculating biomass. Ali et al. (2022) reported that the diameter of the stem is an accurate predictor of biomass. Based on the results obtained in past studies estimating shrub biomass, crown area was considered a reliable predictor of shrub biomass by Maraseni et al. (2005) and McGinnis et al. (2010).

## Conclusion

The growth of *C. procera* and biomass accumulation differ significantly depending on elevation gradient. In this research, the growth performance of *C. procera* was good in terms of plant height, diameter, and crown volume. The urban soil sample at high altitude outperformed the rural area having low altitude and roadside soil with medium altitude groups by a large margin. Differential growth and biomass accumulation data suggest that varied site characteristics impact productivity. The Euclidean distance and the three stand factors of slope direction, elevation (318 ~ 1476 m), and slope gradient was used in this investigation. Compared to the rural and roadside regions, the urban area had the greatest growth performance and biomass accumulation of *C. procera*, indicating that the species is well suited to the soil types of the area. This results suggests that the soil in the region is conducive to *C. procera* development.

## Data availability statement

The original contributions presented in the study are included in the article/supplementary material. Further inquiries can be directed to the corresponding authors.

## Author contributions

S, NK, and HA conducted and analysed the experiments and compiled the data; S and NK wrote the manuscript; S, NK, MO, and MA-M designed and supervised experiments; MA-M, contributed to data analysis; all authors read and approved the manuscript.
